# Genestrip: exact and efficient read classification for selected groups of organisms

**DOI:** 10.1186/s12859-026-06512-1

**Published:** 2026-06-16

**Authors:** Daniel Pfeifer, Markus Graf, Clas Rurik

**Affiliations:** 1 IT Faculty, Heilbronn University, Max-Planck-Str. 39, 74081 Heilbronn, Baden-Württemberg Germany; 2 codeclap, Kokhaven 20, 6230 Rødekro, Denmark

**Keywords:** Metagenomics, *k*-Mer counting, Read classification, Small *k*-mer database, High precision, High recall, High performance

## Abstract

****Background**:**

The consumption of main memory resources is a significant burden in *k*-mer-based metagenomic analysis when creating related databases but also when performing (unique) *k*-mer-counting and read classification. Genestrip addresses this issue by focusing on small but freely configurable groups of organisms. Regarding the selected organisms, Genestrip produces *k*-mer databases and results comparable to those of KrakenUniq but at a fraction of its required memory resources. Our tool ensures that during database generation, the most suitable lowest common ancestor taxon is assigned for each stored *k*-mer by *also considering genomes of organisms whose* *k-mers are not included in the database*. This enables read analysis with high precision and recall for the organisms of interest.

****Results**:**

We assess the correctness, usefulness and performance of Genestrip in different contexts and show that it indeed ascertains high quality read classifications for organisms whose genomes are included in a corresponding database. Our example databases comprise millions to a few billions of *k*-mers covering a dozen to a few thousands of species and lend themselves to usage in tick surveillance, medical diagnostics or agriculture. All databases were generated on a regular PC within hours, and related analysis performance was competitive to highly favorable. The deliberate focus on a particular set of genera or species allows for more genomes to be included from related organisms while the resulting databases remain small. Since *k*-mer compression becomes unnecessary, false positives emerging from related information loss are entirely avoided. We exemplify that such small but deep databases tend to improve recall during read classification while sustaining high precision.

****Conclusions**:**

Due to Genestrip’s particular way of updating the *k*-mers’ lowest common ancestor taxa, both database creation and fastq file analysis can be realized with little memory and with favorable runtimes as well as high classification quality. So both, database creation *and* read classification may be performed even on regular PCs. Genestrip’s qualities empower users to flexibly design, build and use small *k*-mer databases for their own needs with potentially deep genomic coverage.

## Background

With the availability of low-cost and small-scale sequencing devices, metagenomic analysis will likely replace many established technologies for the detection and surveillance of specific groups of microorganisms. Related applications may affect various areas such as farming, ecology, food processing and medicine. While sequencing devices have become ready for ubiquitous use, software tools for metagenomics still tend to be resource intensive and too hard to use for end users.

To analyze large sets of reads, classifications tools based on *k*-mer matching and counting such as Kraken 1 [1], Kraken 2 [2], KrakenUniq [3], Ganon [4, 5] and others are at hand.

With regard to pathogen detection, KrakenUniq is particularly suited because of its very low false positive rate but also because of its efficiency and high sensitivity [3]. In order to avoid false positive classifications of reads, KrakenUniq resorts to very large databases, containing billions of encoded but uncompressed *k*-mers along with their respective taxa. A database is usually loaded entirely into main memory and often consumes tens of gigabytes of space. This requirement mandates specialized and expensive compute servers with very large main memory. In recognition of this problem, KrakenUniq has been extended for database processing in chunks at read analysis time [6]. However, database creation still mandates ultralarge main memory: For example, in 2018 the production of a database for Kraken 1 and, equivalently, KrakenUniq that just covers the bacterial genomes from RefSeq [7] “required ca. 2.5 TB of RAM and ca. 11 days” on a high-performance computer [8]. But since then, RefSeq’s number of prokaryotic genomes alone has increased by a factor of 5.8 to over 48 Mio [9]. Therefore, related organisms are often not covered below the species rank and also genomes below the quality level “complete genome” are usually not included in a corresponding database.

Other *k*-mer based classification tools rely on a variety of approaches to compress the *k*-mers stored in a database. E.g., Kraken 2 offers more memory efficient *k*-mer databases than KrakenUniq but *it is not a suitable alternative in fields such as medical diagnostics where precision of read classification is paramount* [6]. This is because in comparison with Kraken 1 and KrakenUniq, Kraken 2 produces smaller databases, but it stores *k*-mers under loss of information, therefore facilitating false positive *k*-mer counts [2, 6]. Many other recent *k*-mer analysis tools such as Ganon follow this trend and trade database compression against potentially false positive *k*-mer matches [4, 5].

Our contribution pursues an alternative idea by generating small databases that focus on organisms of interest in the context of a specific application scenario. Due to this focus, *k*-mer compression becomes unnecessary. In the course of this study, we show that the approach may lead to higher efficiency and analysis quality in comparison with other *k*-mer based classification tools. On the one hand, we tested Genestrip against KrakenUniq because our tool aims to provide a similar *k*-mer based functionality. On the other hand, we included Kraken 2 and Ganon (Version 2.1) in our evaluations as representatives of *k*-mer compressing systems.

In the results section of this paper, we first assess Genestrip’s correctness via a detailed comparison of analogous analysis results obtained via KrakenUniq (“Correctness” section). Afterwards, we compare classification quality between KrakenUniq, Kraken 2, Ganon and Genestrip using broad coverage, viral databases and simulated viral sequencing data (“Small databases on simulated data” section). We also demonstrate the effects of small databases on real-world data when applying them with regard to human-related viruses under each of the four tools (“Small databases on real-world data” section). As a practical example, we highlight a Genestrip database aiming at tick-borne bacterial pathogens (“Analysis databases” section). We compare classification quality between that Genestrip database and typical broad coverage databases associated with KrakenUniq, Kraken 2 and Ganon (“Analysis of simulated data” section). For this purpose we use realistic, simulated data that closely imitates real-world sequencing data from tick analysis. We complete the classification quality assessment with a brief explorative analysis of real-world tick analysis data. To demonstrate Genestrip’s runtime performance, we compare read analysis speed and related memory consumption between all four tools named above under three scenarios (“Read classification” section). Moreover, Genestrip’s database generation performance is examined via several small example databases designed with real-world use cases in mind (“Database generation” section). Together these databases cover six categories from RefSeq including “Bacterial”, “Fungi” and “Invertebrate”.

### Approach

If only a small group of a few dozen genera or a few 100 species is under consideration for metagenomic analysis, then it may seem promising to simply *generate a small database that contains just the* *k-mers from respective genomes*. *However, without additional measures, this very likely leads to many false positives*: The reason for this is that owing to the evolutionary relationship of organisms, *a large fraction of* *k-mers that belong to the genome of a species are unspecific* in the sense that they can as well be found in the genomes other species or genera not considered for analysis. Thus, when using too small a database, a read may be falsely assigned to a species as it may consist of *k*-mers that *also* belong to another species’ genome not included in the database. In order to enable smaller databases without false positives emerging from this problem, we introduce Genestrip, as new metagenomic software tool supporting (unique) *k*-mer counting and read classification.

In principle, Genestrip employs a *two-phase* database generation process: During the *entry phase*, all *k*-mers of genomes from selected organisms are entered into the database on the basis of RefSeq [7] and optionally GenBank [10] as well as additional sources. During the *update phase*, all stored *k*-mers from the database are checked against the genomes of *all* other organisms from RefSeq belonging to the same category.^1^ So, if a stored *k*-mer also occurs in another organism which is not part of the database, the *k*-mer’s taxon still gets updated in the database and the lowest common ancestor’s taxon (known as LCA) is entered instead [8]. This prevents a *k*-mer from being falsely associated with one of the selected organisms given that it is too unspecific.

Based on this basic approach, Genestrip achieves a low memory footprint during database creation as well as at read analysis time. Moreover, it offers competitive to highly favorable runtimes for *k*-mer matching and read classification. Together, these qualities empower users to create and use respective databases on machines with regular main memory size. Genestrip’s *k*-mer counting results are similar to those of KrakenUniq [3] with respect to the selected organisms. Genestrip also supports unique *k*-mer counting with exact counts and with low memory overhead. It is written in Java, has a small code base and enables command-line-based as well as API-based usage. Via the Java Virtual Machine, it can be executed on modern operating systems including Linux, Windows and macOS.

## Implementation

### System structure

Genestrip’s database generation mandates the setting of groups of organisms whose genomes will be exclusively represented in a resulting database. Users choose the desired genera, species or strains via a configuration file by listing the corresponding taxa. If higher ranked taxa are entered, then by default, *all* of the descending taxa will be included and *k*-mers of their genomes will be added to the database accordingly.

Genestrip generates a database for a selected group of organisms primarily via genomes from RefSeq [7] but genomes from Genbank and additional genomic files may be included on top or instead. By default, related genomic files are only downloaded once to a common folder and shared when further Genestrip databases are to be generated.

To better handle the complexity of the generation process, it employs a goal-oriented approach similar to Make [11]: Upon access, each goal returns a file or an object in main memory. A goal is executed only if its result is not yet available. I.e., in case of a file goal, the system first checks whether the corresponding file is present and only if it is missing, the corresponding goal is executed to produce the file.

Goal execution may involve the access of other goals which in turn, may trigger their execution. Respective goal dependencies are explicitly specified in terms of a directed acyclic graph. Details on the system’s goals and the goal graph can be found in Sect. 1 of the Appendix.

### Database generation

Genestrip stores a database primarily as a large array *L* of sorted long values. Each long value encodes a *k*-mer from the genome of a selected organism. The default for *k* is 31 but smaller values and $k=32$ are possible. In order to handle reverse complements of reads later at read analysis time, stored *k*-mers are standardized in the following way: Each *k*-mer is encoded twice as a long value—once in its canonical version and once via its reverse complement—but only the larger of the two encoded values is stored in the database. A second array *T* with the same entry size as *L* encodes taxa for each *k*-mer in *L* at corresponding positions. At read analysis time, lookups in *L* are performed via a binary search.

All parts of the NCBI taxonomy tree [12] required at analysis time are also included in the database. The taxonomy extract comprises entries for the selected organisms and all of their ancestors. At analysis time, a bit vector with the same entry size as *L* indicates which *k*-mers from *L* were found at least once. This is sufficient for exact unique *k*-mer counting and is space-efficient, as it requires only about 1.6% of additional main memory with respect to *L*.

Genestrip employs one Bloom filter [13] at *database generation time* with a very low false positive probability (FPP). While the database gets populated, it tracks the already stored *k*-mers to avoid duplicates. Since *L* is not sorted at that point in time, the filter offers an efficient containment check.^2^

A so-called Blocked Bloom filter [14] with a rather high FPP of almost 1% gets constructed after *L* got sorted. It is exclusively used for performance optimization *at read classification time*:^3^ This filter is consulted prior to a *k*-mer lookup in *L* to check whether the *k*-mer might be in *L*. *A negative result avoids a considerably more expensive binary search in* *L* *and therefore saves overall execution cost.* We compared the average access time to look up a random *k*-mer in Genestrip’s *k*-mer store with and without the Blocked Bloom filter being activated: Depending on the size of the database the filter brought an improvement of about factor 9 for very small databases to roughly factor 11 for larger databases. So, at read analysis time, this filter merely improves efficiency but does not cause false positives, since a hit is always backed by *L*. Employing the filter pays off because in practice, many *k*-mers from reads are likely not stored in a typical database.^4^

Regarding database generation, additional genomes can be manually added either to create deeper databases or, else, to filter out contaminating sequences that are mistakenly included in the genome of an organism of interest. For example, Breitwieser et al. [16] reported that human DNA fragments often contaminate bacterial genomes for various reasons whereas Lu and Salzberg [17] discussed a pipeline to remove contaminants from genomes of eukaryotic pathogens. With Genestrip, *k*-mers from contaminating sequences can be identified during the update phase as introduced in “Approach” section: For example, the human genome can be included via configuration in the update phase only; thus, a *k*-mer from the human genome that is also found in the contaminated genome will be pushed to the lowest common ancestor taxon of both species. Still, as the human genome is employed during the update phase only, a resulting database does not include *k*-mers from the human genome (other than those from contaminating sequences). This ensures a small-sized database.

Of note, Genestrip allows for the exclusion of *k*-mers with low complexity known as genomic dust [18]: As part of the entry phase, each *k*-mers’s repetitiveness in terms of base pairs is measured via a fibonacci-based function. If the corresponding value exceeds a configurable threshold then the *k*-mer is dropped. Section 2 of the Appendix presents our dust function in detail.

### Read classification

The goal match loads a database and performs the read classification on fastq files. The principal classification algorithm is similar to that from Kraken 1 [1] and equivalently KrakenUniq. When iterating over a read’s *k*-mers, each *k*-mer is encoded as a long—once by considering its canonical version and once via its reverse complement.^5^ For efficiency, only the larger of the two encoded values is looked up in the database. This is sufficient since, if at all, then only just the larger of two values got stored in the first place during database generation (see “Database generation” section). Genestrip implements a multithreading enabled classification procedure following a producer consumer pattern, where a producer thread reads a fastq file and puts reads into an in-memory queue while consumer threads pick reads from the queue in order to classify them. Three types of error thresholds can be configured for read classification:

1. The ratio or number of *k*-mers per read that must at least be found in the database and
2. the ratio or number of *k*-mers per read that must be consistent with the read’s designated class taxon. A *k*-mer is consistent with a read’s class taxon if the *k*-mer’s taxon as stored in the database is the same or if it is a taxonomic ancestor of the class taxon.
3. Moreover, the classification threshold minKMersForClass supports the reduction of potential false positive classifications. It can be set to adjust the minimal sum of *k*-mers under taxon *t* required for a read to be classified to *t*. I.e., given a read *r* and taxon $t_1$ on the genus rank with two *k*-mers in *r* and taxon $t_2$ subordinate to $t_1$ on the species rank with one *k*-mer in *r*. Furthermore, *r* shall contain no other *k*-mers matching any taxons. Then, if minKMersForClass=2, *r* would *not* be classified to $t_1$ but to $t_2$ instead since the single *k*-mer under $t_1$ is below the threshold but the sum of three *k*-mers under $t_2$ is sufficient.

The thresholds allow for playing off recall against precision in light of potentially high error rates of some sequencing devices [19, 20]. For all the experiments throughout this paper, the first two thresholds were left at the default of one *k*-mer which is the most sensitive level.

### Analysis reports

The read classification via the goal match produces analysis reports in CSV file format with a comprehensive set of statistical values. We use the following notation to describe them: Let $k_{t,f,d}$ be the number of *k*-mers for taxon *t* found in fastq file *f* with respect to database *d* and let $u_{t,f,d}$ be the corresponding number of unique *k*-mers. Similarly, let $u_{t,d}$ be the number of (unique) *k*-mers for *t* stored in *d*.

Table 1 describes the most important reported columns. Additional columns exist to accumulate *k*-mer counts and read counts etc. upward to ancestors in the taxonomy. We also track the standard deviations of avg. contig length, avg. read length, mean class error and other columns wherever suitable. Moreover, normalized values derived from the original values by dividing by $u_{t,d}$ are included where suitable. Normalized values aim to reduce counting bias due to a high or low representation of a taxon *t* in terms of its stored *k*-mers $u_{t,d}$.

**Table 1:** Extract of columns from Genestrip’s read analysis reports

Column name	Description
taxid	The class taxon *t*
reads	The number of reads classified with respect to *t*
kmers from reads	The number of *k*-mers from reads for *t* that are consistent with *t*
kmers	$k_{t,f,d}$
unique kmers	$u_{t,d}$
avg. contig length	The average length of contiguous sequences of *k*-mers that are specific to *t*’s genome(s) as represented in *d*
max contig length	The maximum length of all contiguous sequences of *k*-mers that are specific to *t*’s genome(s)
avg. read length	The average length of reads for *t* in base pairs
db coverage	$u_{t,f} / u_{t,d}$
mean class error	The mean ratio of *k*-mers from a read *r* for *t*, where those *k*-mers are not consistent with *t* per *r*’s total number of *k*-mers
exp. unique kmers	The expected unique *k*-mers $E(k_{t,f,d},u_{t,d})$ (see Sect. 3 of the Appendix for details)
unique kmers/exp.	The *k*-mer consistency ratio $r_{t,f,d}$ (see Sect. 3 of the Appendix for details)

### Example usage

We give a brief impression on how to use Genestrip from the Unix command line.^6^ The installation includes a sample project human_virus. After building Genestrip, you may call
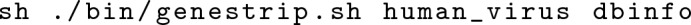
from the installation directory to generate the human_virus database and also, to create a CSV file with basic information about the database content. As stated in “System structure” section, Genestrip follows a goal-oriented approach in order to create any result files. Therefore, when generating the human_virus database, Genestrip will automatically

1. download the NCBI taxonomy and unzip it to ./data/common, if not yet present,
2. download the RefSeq release catalog to ./data/common/refseq, if not yet present,
3. download all virus related RefSeq fna files to ./data/commmon/refseq (which is currently just a single g-zipped file), if not yet present,
4. perform several follow-up goals, until the database file human_virus_db.zip is finally made under ./data/projects/human_virus/db and
5. create a CSV file human_virus_dbinfo.csv under ./data/projects/human_virus/csv, which contains basic information about the database, i.e. the number of *k*-mers stored per taxon.

The generated database comprises *k*-mers for all viruses whose taxons are listed in the configuration file ./data/project/human_virus/taxids.txt.

To try out classification, the installation comes with the small fastq file sample.fastq.gz in ./data/projects/human_virus/fastq. One may trigger its analysis via
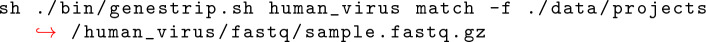


The resulting CSV file named human_virus_match_sample.csv can then be found under ./data/projects/human_virus/csv.

## Results

### Correctness

To assess Genestrip’s correctness with regard to *k*-mer counting and read classification, we compared example databases and respective analysis results directly and in detail with those from KrakenUniq. As part of our test procedure, we created database contents under *both* systems that were identical in terms of their genomic basis and their stored *k*-mers. We then applied the databases to suitable fastq files in order to compare the results between both systems.^7^ Highly similar results from this check mutually solidify the correctness of both systems since they had been developed completely independently. (In addition, Genestrip’s correctness is also supported via extensive automated functional tests with high test coverage as part of its code base.)

We chose all RefSeq genomes from the category “Viral” for the two databases. The latter turned out to be completely identical in terms of the number of *k*-mers stored per taxon. In the following, we name the two databases cv_ku and cv_g with regard to KrakenUniq and Genestrip, respectively.

We applied cv_ku and cv_g to a simulated fastq file extracted from [21] where the file’s reads were used to benchmark metagenomic pipelines aimed at the discovery of new viruses. The file contains 61,550 long reads extracted from 6155 viral standard genomes from RefSeq [21]. Furthermore, we generated simulated fastq files based on all RefSeq genomes from the category “Viral” via the tool InSilicoSeq [22]: We applied the respective Illumina generation models “MiSeq” and “HiSeq” to produce two pairs of fastq files with one million reads per pair. Then, we analyzed these files via cv_ku and cv_g as well. In all of the cases the *k*-mer counts per taxon were identical between KrakenUniq and Genestrip and the classified read counts were also identical.

As real-world examples, we applied the two databases to short-read fastq files from DNA-sequencing human saliva of five different donors. Table 2 shows basic statistics of these files as downloaded from the Sequence Read Archive [23]. Again, we achieved completely identical *k*-mer and read counts between KrakenUniq and Genestrip. Details on some of these comparisons can be found in Sect. 4 of the Appendix.

**Table 2:** Real-world fastq files from DNA-sequencing five human saliva samples as used throughout the evaluation (downloaded from the sequence read archive [23])

#	Base file name	Reads	Avg. BPs per read
1	SRR5571985	812,085,208	101
2	ERR1395613	900,709,176	191
3	SRR5571991	980,879,835	276
4	ERR1395610	824,479,570	429
5	SRR5571990	605,561,636	685

As previously stated, this perfect agreement mutually solidifies the correctness of both systems, but obviously, it does not yet highlight the advantages of Genestrip when creating and using small databases for selected groups of organisms.

### Classification via broad coverage databases

As a baseline, we tested read classification quality using all of the simulated data introduced in “Correctness” section. With the ground truth known, this allowed for comparing precision and recall across different *k*-mer-based analysis tools.

Regarding the databases, we reused cv_ku and cv_g from “Correctness” section and generated analogous databases for Kraken 2 and Ganon, respectively. Ganon depends on an important hyper-parameter max-fp that controls the false positive probability (FPP) for matching *k*-mers and must be set at database generation time. (A lowered FPP incurs a larger database.) We used the default value of 0.001 (combined with --filter-type hibf) which is documented as highly sensitive in Ganon’s online manual.^8^

All analysis tools but KrakenUniq offer classification time options to trade sensitivity against confidence. As listed in Table 3, we examined two variants for each tool—a variant with higher sensitivity indicated by “(S)” (which is the default setting for Kraken 2 and Genestrip) and a variant with higher confidence indicated by “(C)”. Ganon performs multiple classifications for reads—typically in less than 15% of the cases. To enable a reasonably fair comparison, we computed the lowest common ancestor when Ganon produced multiple class taxons for a read and used the outcome in the evaluations.

**Table 3:** Classification time settings for each tool: a higher sensitivity and a higher confidence variant are examined in most of the experiments from “Results” section

System variant	Descripton	Option settings
KrakenUniq	High sensitivity (default)	None
Kraken 2 (S)	High sensitivity (default)	None
Kraken 2 (C)	High confidence	--confidence 0.5
Ganon (S)	High sensitivity	--rel-cutoff 0 --rel-filter 0
Ganon (C)	High confidence (default)	None
Genestrip (S)	High sensitivity (default)	None
Genestrip (C)	High confidence	minKMersForClass=4

KrakenUniq does not offer related options and therefore, its variant name carries no “(S)” or “(C)”

Table 4 shows that most systems perform within reason except for the configuration “Kraken 2 (C)”. Irrespective of its configuration, Ganon appears to be sensitive to the higher error rates in “MiSeq” as opposed to “HiSeq”.

**Table 4:** Read classification quality for different simulated fastq files analyzed with KrakenUniq, Kraken 2, Ganon and Genestrip

System	Data	Species			Genus		
		Prec.	Recall	F$_1$	Prec.	Recall	F$_1$
KrakenUniq	Extr. from [21]	0.95	0.95	0.95	1.00	1.00	1.00
Kraken 2 (S)	Extr. from [21]	0.95	0.95	0.95	1.00	1.00	1.00
Kraken 2 (C)	Extr. from [21]	0.71	0.71	0.71	0.95	0.95	0.95
Ganon (S)	Extr. from [21]	0.97	0.97	0.97	1.00	1.00	1.00
Ganon (C)	Extr. from [21]	0.96	0.96	0.96	1.00	1.00	1.00
Genestrip (S)	Extr. from [21]	0.95	0.95	0.95	1.00	1.00	1.00
Genestrip (C)	Extr. from [21]	0.95	0.95	0.95	1.00	1.00	1.00
KrakenUniq	MiSeq	0.91	0.91	0.91	0.99	0.99	0.99
Kraken 2 (S)	MiSeq	0.91	0.91	0.91	0.99	0.99	0.99
Kraken 2 (C)	MiSeq	0.77	0.76	0.76	0.96	0.93	0.94
Ganon (S)	MiSeq	0.92	0.92	0.92	0.99	0.99	0.99
Ganon (C)	MiSeq	0.92	0.77	0.83	0.99	0.83	0.90
Genestrip (S)	MiSeq	0.91	0.91	0.91	0.99	0.99	0.99
Genestrip (C)	MiSeq	0.91	0.91	0.91	0.99	0.99	0.99
KrakenUniq	HiSeq	0.88	0.88	0.88	0.98	0.98	0.98
Kraken 2 (S)	HiSeq	0.88	0.88	0.88	0.98	0.98	0.98
Kraken 2 (C)	HiSeq	0.79	0.78	0.79	0.96	0.95	0.95
Ganon (S)	HiSeq	0.89	0.89	0.89	0.98	0.98	0.98
Ganon (C)	HiSeq	0.88	0.83	0.86	0.98	0.93	0.96
Genestrip (S)	HiSeq	0.88	0.88	0.88	0.98	0.98	0.98
Genestrip (C)	HiSeq	0.88	0.88	0.88	0.98	0.98	0.98

Regarding KrakenUniq and Genestrip the viral databases cv_ku and cv_g from “Correctness” section were used. Similar (unnamed) viral databases were generated for Kraken 2 und Ganon. The systems’ adaptions for “(S)” and “(C)” are based on Table 3

### Classification via small databases

The following results highlight the importance of the update phase according to “Approach” section in the context of small databases.

#### Human virus versus complete viral databases

As a first example, we created two small viral databases based on RefSeq covering *human viruses only*,^9^ again under both KrakenUniq and Genestrip. In the following, we name the two databases hv_ku and hv_g with regard to KrakenUniq and Genestrip, respectively. We again assured that the databases were identical in terms of their genomic basis and their stored *k*-mers. In addition, we generated analogous human virus databases for Kraken 2 and Ganon with the same configurations for Ganon as described in “Classification via broad coverage databases” section.

**Fig. 1:**
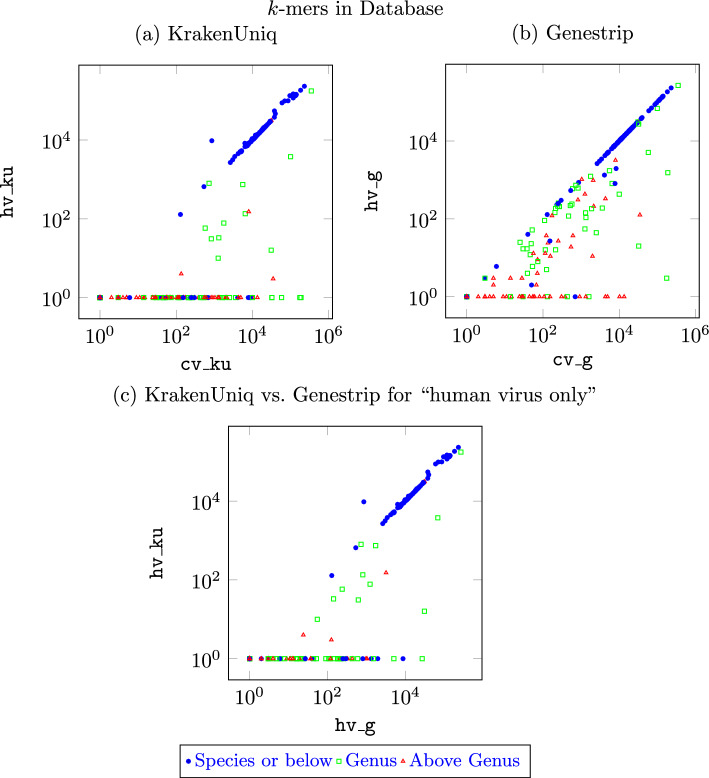
Scatter plots of *k*-mers per taxon in a complete viral database versus a “human virus only” database both from RefSeq created via **a** KrakenUniq and **b** Genestrip (based on a logarithmic scale with 1 added to each value to enable the representation of zero-counts). In addition, **c** directly compares the *k*-mer counts of the two “human virus only” databases hv_ku and hv_g

Figure 1 compares the viral database contents for the databases cv_ku and cv_g from “Correctness” section against the respective “human virus only” database for (a) KrakenUniq and (b) Genestrip. In addition, (c) directly compares the *k*-mer counts of the two “human virus only” databases hv_ku and hv_g. The plots each depict data points of the 422 taxa referenced in at least one of the two corresponding databases.

Regarding Genestrip’s databases from Fig. 1b, nearly all *k*-mer counts on the species rank and below are identical per taxon as an effect of the update phase (that we discussed in “Background” section). This is an important precondition met by Genestrip’s hv_g database to avoid false positives at read classification time. Nevertheless, data points from higher taxonomic ranks scatter because the complete viral database cv_g contains *k*-mers from many more non-human viruses, and these *k*-mers were pushed to higher ranks due to the lowest common ancestor update. Obviously, these additional *k*-mers are not required to classify reads accurately with respect to human viruses. The database hv_g contains only 3,865,987 *k*-mers with a disk size of approximately 32 MB, whereas cv_g has as disk size of 3.1 GB with 98 times as many *k*-mers.

KrakenUniq’s corresponding *k*-mer counts from Fig. 1a are not identical for the species rank and below. Although visually not well obvious in (a), this accounts for more than 150 scattering data points on the species rank and below. These differences offer the potential for more error and in particular false positives at read classification time as will be exemplified next. The plots (a) and (c) look similar because with regard to human virus taxa, hv_g is similar to cv_ku and cv_g.

#### Small databases on simulated data

We applied the “human virus only” databases to the “MiSeq” and “HiSeq” data from “Correctness” section and again measured precision and recall but with respect to human viruses only.^10^ So, this evaluation examines the quality of a small database on simulated data where the ground truth is known but with a focus on just the genomes and taxons used to create the small databases. Since about 98% of the reads in “MiSeq” and “HiSeq” data are from non-human-related viruses, they constitute noise in this context.

Table 5 shows that all systems still perform within reason on the simulated data but with Genestrip clearly in the lead.

**Table 5:** Read classification quality for simulated fastq files from “Correctness” section analyzed with KrakenUniq, Kraken 2, Ganon and Genestrip using respective “human virus only” databases and with an evaluation focus on human viruses only

System	Data	Species			Genus		
		Prec.	Recall	F$_1$	Prec.	Recall	F$_1$
KrakenUniq	MiSeq	0.71	0.96	0.82	0.71	0.96	0.82
Kraken 2 (S)	MiSeq	0.70	0.96	0.81	0.70	0.96	0.81
Kraken 2 (C)	MiSeq	0.95	0.91	0.93	0.95	0.91	0.93
Ganon (S)	MiSeq	0.37	0.96	0.53	0.37	0.96	0.53
Ganon (C)	MiSeq	0.95	0.80	0.87	0.95	0.80	0.87
Genestrip (S)	MiSeq	0.95	0.96	0.95	0.95	0.96	0.96
Genestrip (C)	MiSeq	0.95	0.96	0.96	0.96	0.96	0.96
KrakenUniq	HiSeq	0.82	0.97	0.89	0.82	0.97	0.89
Kraken 2 (S)	HiSeq	0.82	0.97	0.89	0.82	0.97	0.89
Kraken 2 (C)	HiSeq	0.96	0.95	0.95	0.96	0.95	0.95
Ganon (S)	HiSeq	0.68	0.97	0.80	0.68	0.98	0.81
Ganon (C)	HiSeq	0.96	0.93	0.94	0.96	0.93	0.95
Genestrip (S)	HiSeq	0.97	0.97	0.97	0.98	0.98	0.98
Genestrip (C)	HiSeq	0.97	0.97	0.97	0.98	0.98	0.98

The systems’ adaptions for “(S)” and “(C)” are based on Table 3

#### Small databases on real-world data

As real-world examples, we first applied the “human virus only” databases to the short-read fastq files from Table 2 (and introduced in “Correctness” section).

**Fig. 2:**
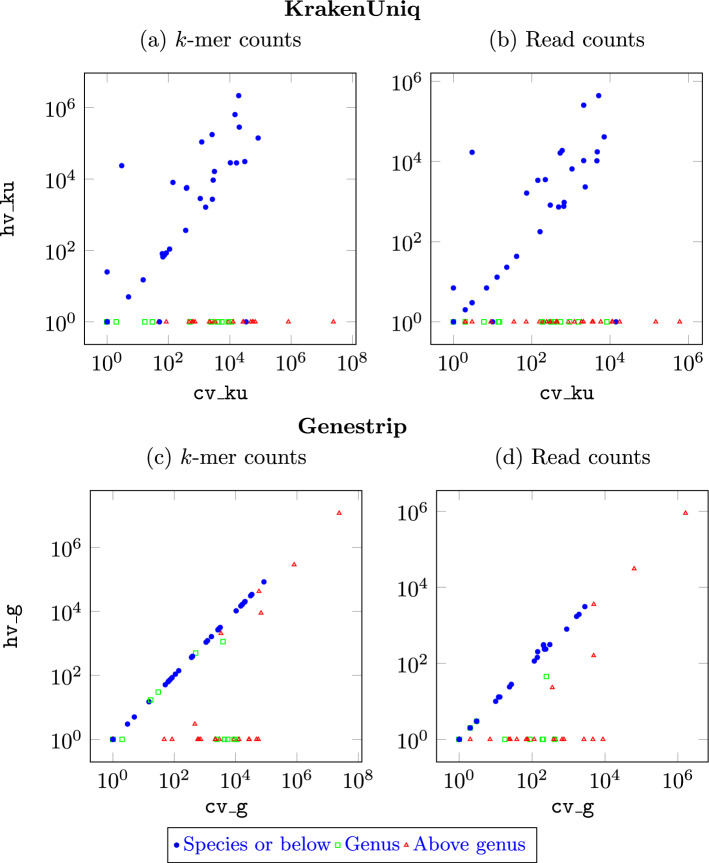
Scatter plots of *k*-mer counts (**a**, **c**) and read counts (**b**, **d**) for the human saliva fastq file #3 from Table 2 analyzed with KrakenUniq (**a**, **b**) and Genestrip (**c**, **d**) using the complete viral databases versus the “human virus only” databases, as both shown in Fig. 1 (based on a logarithmic scale with 1 added to each value to enable the representation of zero-counts)

Figure 2a and b shows scatter plots of *k*-mer counts and read counts from KrakenUniq on a logarithmic scale for the database cv_ku versus hv_ku when applied to the human saliva fastq file #3 from Table 2. Figure 2c and d shows corresponding results with respect to the Genestrip databases cv_g versus hv_g: While the small database under KrakenUniq led to massive overcounts of *k*-mers for the rank species and below, this did not happen under Genestrip. Moreover, KrakenUniq’s overcounting of *k*-mers strongly influenced the counts of classified reads. Regarding Genestrip, the read counts on the species level and below are affected only by differing *k*-mer counts that occurred on the genus level and above. However, the impact remains small. The results for the other human saliva fastq files are similar (not shown).

We also measured a relative read classification quality for all of the five human saliva fastq files from Table 2 as well as for simulated fastq files from “Correctness” section by comparing the “human virus only” database against the corresponding complete viral database under KrakenUniq, Kraken 2, Ganon and Genestrip—again with an evaluation focus on human viruses only. Although no ground truth is available for the human saliva fastq files, this comparison tells *how much classification quality suffers under each analysis tool when the corresponding database is filled with just the genomes of interest—i.e., the genomes of human viruses only instead of all viral genomes*. The result represents a relative factorial change where an approximate baseline for the simulated data is given by Tables 4 and by Table 4 of the Appendix.

Table 6 shows the results for simulated data and fastq file #3 from Table 2. The results for the other four human saliva fastq files are similar and supplied in Table 5 of the Appendix. While KrakenUniq and Ganon still achieve a mediocre to good F$_1$-score on simulated data, their precision is insufficient regarding the human saliva fastq files. This is due to massive false positive classifications of reads, and is a consequence of a missing consideration of non-human-related viral genomes during database generation. Although speculative, we assume that the real-world data has a different error profile than the simulated data, which may contribute to the stark difference. Also, reads belonging to host DNA may cause critical noise. “Kraken 2 (C)” offers reasonable results, but “Genestrip (C)” starts from a better baseline in Table 4, and its human virus database holds up because it does consider non-human-related viral genomes as part of the update phase according to “Approach” section.

**Table 6:** Read classification quality for simulated fastq files from “Correctness” section as well as the real-world fastq file #3 from Table 2 analyzed with KrakenUniq, Kraken 2, Ganon and Genestrip with an evaluation focus on human viruses only

System	Data	Species			Genus		
		Prec.	Recall	F$_1$	Prec.	Recall	F$_1$
KrakenUniq	MiSeq	0.71	0.96	0.82	0.71	0.96	0.82
Kraken 2 (S)	MiSeq	0.70	0.96	0.81	0.70	0.96	0.81
Kraken 2 (C)	MiSeq	0.93	0.95	0.94	0.93	0.95	0.94
Ganon (S)	MiSeq	0.37	0.96	0.53	0.37	0.96	0.53
Ganon (C)	MiSeq	0.95	0.96	0.95	0.95	0.96	0.95
Genestrip (S)	MiSeq	0.95	0.96	0.96	0.95	0.96	0.96
Genestrip (C)	MiSeq	0.96	0.96	0.96	0.96	0.96	0.96
KrakenUniq	HiSeq	0.82	0.98	0.89	0.82	0.98	0.89
Kraken 2 (S)	HiSeq	0.81	0.98	0.89	0.81	0.98	0.89
Kraken 2 (C)	HiSeq	0.94	0.98	0.96	0.94	0.98	0.96
Ganon (S)	HiSeq	0.68	0.98	0.80	0.68	0.98	0.80
Ganon (C)	HiSeq	0.96	0.98	0.97	0.96	0.98	0.97
Genestrip (S)	HiSeq	0.98	0.98	0.98	0.98	0.98	0.98
Genestrip (C)	HiSeq	0.98	0.98	0.98	0.98	0.98	0.98
KrakenUniq	#3 from Table 2	0.04	0.91	0.07	0.04	0.91	0.07
Kraken 2 (S)	#3 from Table 2	0.03	0.65	0.06	0.03	0.65	0.06
Kraken 2 (C)	#3 from Table 2	0.40	1.00	0.57	0.40	1.00	0.57
Ganon (S)	#3 from Table 2	0.02	0.69	0.04	0.02	0.70	0.04
Ganon (C)	#3 from Table 2	0.25	1.00	0.40	0.25	1.00	0.40
Genestrip (S)	#3 from Table 2	0.50	1.00	0.67	0.50	1.00	0.67
Genestrip (C)	#3 from Table 2	0.93	1.00	0.96	0.93	1.00	0.96

The evaluation compares the classifications when using a “human virus only” database against each system’s own classifications obtained when using the corresponding complete viral database from “Classification via broad coverage databases” section. The systems’ adaptions for “(S)” and “(C)” are based on Table 3

### Example: Tick-borne bacterial pathogens

This section presents the detection of tick-related bacterial pathogens as an example application of Genestrip. Once again, we compare our tool against KrakenUniq, Kraken 2 and Ganon and reuse or generate standard databases available for the latter systems. Regarding Genestrip, we employ a relatively small database tb with high genomic coverage for the organisms of interest.

An envisioned application is tick surveillance via metagenomics according to [25], where the authors applied Nanopore technology to check eight field-collected ticks but back then relied on Kraken 2 to identify corresponding organisms. Due to Huggins et al. [26], Miao et al. [27] and Huggins et al. [28] we also see a use case when screening for tick-borne pathogens in vertebrates potentially including humans.

#### Analysis databases

We discuss the databases used in conjunction with each system but with an emphasis on Genestrip’s database tb.

MicrobialDB [29] for KrakenUniq aims at the diagnosis of infectious diseases using metagenomics and contains about 98 times as many *k*-mers as tb. It has a disk size of 574 GB and covers a broad set of microbials including bacteria and the human genome with contaminants removed [17]. Regarding Kraken 2, we used the latest Standard database from Langmead [29] covering the entire RefSeq category “Bacteria”, the human genome and more with a disk size of 73 GB and with about 18 billion compressed *k*-mers. For Ganon, we generated a database covering all genomes of the category “Bacteria” from RefSeq Release 233 using Ganon’s default FPP (see also “Classification via broad coverage databases” section) which resulted in a database size of 58 GB.

The database tb for Genestrip is based on RefSeq Release 233 and covers all RefSeq genomes under the twelve bacterial genera Bartonella, Rickettsia, Anaplasma, Ehrlichia, Borrelia, Borreliella, Chlamydia, Mycoplasma, Malacoplasma, Mycoplasmoides, Metamycoplasma and Francisella as many included species are pathogenic to humans and known or assumed to occur in ticks [30–34]. To avoid contaminants from human DNA and black-legged tick DNA, we added corresponding genomes to Genestrip’s update phase as explained at the end of “Database generation” section. We also activated the genomic dust filter at moderate sensitivity—e.g., the *k*-mer TTTTTTTTTTTTTCGGTGTTTTTTTGTTACT would have been dropped because its repetitiveness is just above the set threshold.

tb contains about 525 million uncompressed *k*-mers and has a disk size of just 3.91 GB including the accompanying Bloom filter and a suitable extract of the NCBI taxonomy tree. tb has 1.96 times as many *k*-mers stored under the selected genera and below as MicrobialDB. The main reason behind this increase is the general growth of RefSeq’s number of prokaryotic genomes between the generation of MicrobialDB in mid 2023 and the generation of tb [9].

The generation of tb revealed that its included genomes from RefSeq have a high overlap: On average each stored *k*-mer occurred 11 times in these genomes. Only 0.0079% of all *k*-mers considered for storing were dropped as “dust”. The update phase pushed 26.3% of the stored *k*-mers to ranks above genus. 0.027% of the stored *k*-mers had been pushed to the taxon of “cellular organisms” because tick DNA and human DNA were included on purpose during the update phase for the sake of contaminant detection.

#### Analysis of simulated data

To assess classification quality for this case, we first generated ground truth data via the eight real-world fastq files from [25] mentioned above. For this purpose, we employed NanoSim [35, 36], a tool which is capable of generating realistic long reads based on a set of genomes and a sample fastq file used for training. The tool tries to reflect the genomic abundance of organisms from the training fastq file. Moreover, it imitates long read sequencing errors resulting from Nanopore technology via an error model which is also estimated on the basis of the training fastq file.

We supplied NanoSim with all RefSeq genomes from the twelve bacterial genera mentioned in “Analysis databases” section and used each of the eight tick-related real-world fastq files to generate corresponding *simulated* fastq files. The number of reads per simulated file ranges between 116,315 and 1,361,189 with 330,046 reads on average and a mean read length of 3926 BPs.

It is important to know that both, the real-world data and the simulated data are subject to high error in their bases: As measured via “Genestrip (S)”, the mean ratio of *k*-mers that could not be assigned to any taxon in classified reads was 83% and 88%, respectively. Regarding the simulated data, the error cannot be attributed to missing *k*-mers in tb, since the simulated data was generated only via genomes also represented in tb. In fact, the measured error is consistent with a per-base error between 5 and 15% as reported in [37] for Nanopore devices.^11^

We applied the analysis tools with their respective databases to the eight simulated fastq files. Apparently due to high error rates in the data, Ganon did not classify any reads under the variant “Ganon (C)”—hence, we show no results for this case. Instead, we present an additional variant, “Ganon (A)”, where thresholding was completely turned off via the combined options -rel-cutoff 0, --rel-filter 0 and --fpr-query 1e-0 causing all reads to be classified.

Table 7 shows the macro-averaged results for each system. (Detailed results for the first five of the eight simulated fastq files are listed in Table 6 of the Appendix.) Apparently, all analysis tools struggle with the high error from the modeled Nanopore device but Genestrip’s configurations perform far better than the others. We attribute this to the deep genomic coverage of the organisms of interest in tb combined with a loss-less storing of related *k*-mers and Genestrip’s thorough least common ancestor update.

**Table 7:** Macro-averaged read classification quality for simulated data derived from an analysis of bacterial pathogens in ticks [25]

System	Species			Genus		
	Prec.	Recall	F$_1$	Prec.	Recall	F$_1$
KrakenUniq	0.41	0.13	0.18	1.00	0.23	0.34
Kraken 2 (S)	0.54	0.18	0.26	1.00	0.30	0.45
Kraken 2 (C)	0.35	0.00	0.01	1.00	0.01	0.02
Ganon (S)	0.34	0.16	0.21	0.58	0.25	0.34
Ganon (A)	0.17	0.17	0.17	0.26	0.26	0.26
Genestrip (S)	0.60	0.44	0.51	0.78	0.57	0.65
Genestrip (C)	0.58	0.40	0.47	0.76	0.52	0.61

The results are based on eight simulated fastq files generated using NanoSim [35, 36] on the basis of eight corresponding real-world, tick-related fastq files. In “Analysis databases” section describes the databases used and “Analysis of simulated data” section discusses the analyzed data. The systems’ adaptions for “(S)” and “(C)” are based on Table 3; “Ganon (A)” is described in “Analysis of simulated data” section

#### Explorative analysis of tick-related data

In order to investigate the effect of databases with deep genomic coverage, we applied KrakenUniq and Genestrip along with MicrobialDB and tb to the eight *real-world* fastq files from Kipp et al. [25] mentioned above. For this comparison we take advantage of the compatibility of Genestrip and KrakenUniq as established in “Correctness” section.^12^ Then, differences of classification results solely originate in the databases—but as explained in “Analysis databases” section, tb is deeper than MicrobialDB as it has almost twice as many *k*-mers stored for its included genera.

**Fig. 3:**
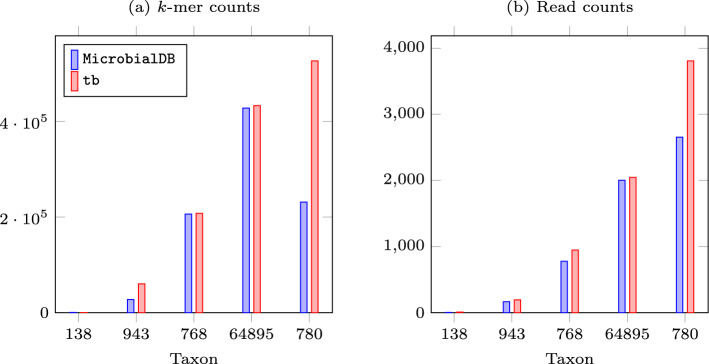
Comparison of *k*-mer counts (**a**) and read counts (**b**) between the database MicrobialDB (based on KrakenUniq) and tb (based on Genestrip). Counts are aggregated up to rank genus for the long read fastq file of “Tick 6” from [25] and restricted to genera included in tb with only the five most frequent genera in terms of *k*-mer counts shown

As an example result, Fig. 3 shows the five most frequent *k*-mer counts and reads counts for “Tick 6” accumulated up to the genus rank. In all cases but “138” (Borrelia), the aggregated counts for the genera under tb are mildly to considerably higher than those achieved with MicrobialDB. We observed similar improvements in terms of *k*-mers and reads with respect to Ehrlichia, Rickettsia and partly to Anaplasma in all of the eight ticks’ fastq files whenever corresponding organisms showed presence via MicrobialDB. The effect is consistent with Genestrip’s higher recall on the simulated data from Table 7.

**Fig. 4:**
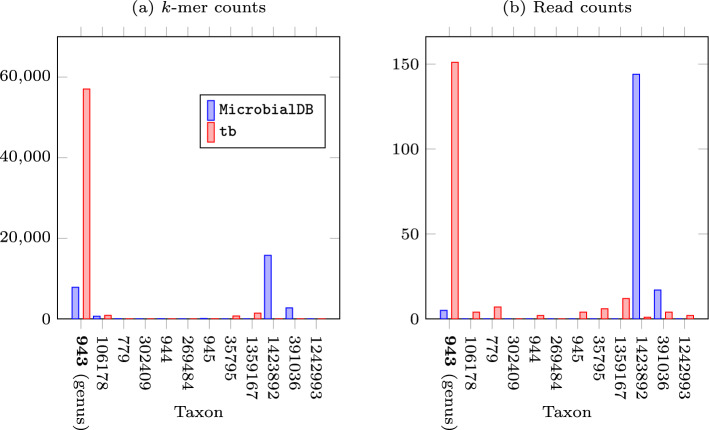
Comparison of *k*-mer counts (**a**) and read counts (**b**) between the database MicrobialDB (based on KrakenUniq) and tb (based on Genestrip) for all taxons under the genus “943” (Ehrlichia) including the genus itself. The counts belong to the long read fastq file of “Tick 6” from [25]—the same file as in Fig. 3

Figure 4 details results for Tick 6 with respect to “943” (Ehrlichia): Interestingly, Genestrip assigns most of the found *k*-mers right to the genus, whereas KrakenUniq produces a peak under strain “1423892” (Ehrlichia muris AS145). The difference originates in the deeper database tb, where in comparison with MicrobialDB, more common *k*-mers were found from a broader range of genomic data under the genus Ehrlichia at tb’s generation time. In turn, Genestrip’s update phase pushed these *k*-mers to the genus rank as its lowest common ancestor. Indeed, the ratio of stored *k*-mers between tb and MicrobialDB is 4.54 for taxon “943” but only 0.24 for “1423892”.

Again, we observed similar results, particularly with respect to Ehrlichia, Rickettsia and partly to Anaplasma, in all of the eight ticks’ fastq files whenever corresponding organisms were present via MicrobialDB. These findings form a general trend when *k*-mer databases with a lowest common ancestor representation become deeper. The effect was first discovered and studied in [8]. Note that this is beneficial since such deeper *k*-mer databases tend to protect read classification from potentially falsely assigning a too low-ranked taxon.

### Performance

As mentioned in “Background” section, Genestrip’s focus is on generating and using potentially small but also deep *k*-mer databases for metagenomic analysis of selected organisms of interest. This implies that users must be empowered to flexibly create new databases—ideally on their own devices. We therefore verify that Genestrip’s database generation performance adheres to this requirement. Moreover, we report on read classification performance in comparison with KrakenUniq, Kraken 2 and Ganon.

The following experiments were all performed on a modern PC with 128 GB of RAM, 10 CPU cores and a 2 TB solid state disk. Although for Genestrip about 32 GB of RAM would have sufficed, we needed the additional RAM for the competing analysis tools.^13^

#### Database generation

Table 8 lists six Genestrip databases used for assessing database generation performance. They refer to several categories and were designed with a potential use in medicine or agriculture in mind. Table 9 reports execution statistics for the generation of these databases from scratch. Execution times and maximum RAM remained moderate in all cases.^14^  

The different execution times in Table 9 depend on the size of the database itself but also on the RefSeq category, where “Bacteria” is the largest of the five ones used with genomes in over 3250 zip files with a total disk size of over 575 GB (in RefSeq Release 233). The zip files must all be screened during each goal from Table 1 of the Appendix but the update phase, i.e., the goal updatedb, dominates the total execution time.

**Table 8:** Background on Genestrip databases used for assessing database generation performance

DB name	Category	Description	Species and below
cv_g	Viral	See “Correctness” section	25,739
hv_g	Viral	See “Classification via small databases” section	225
tb	Bacterial, Plasmid	See “Analysis databases” section	1119
protozoa	Protozoa	Comprehensive collection of protozoans from RefSeq which are pathogenic to humans [38]	108
vineyard	Fungi	Fungal infections from RefSeq and Genbank threatening grapevine plants [39]	11
parasites	Invertebrate	Comprehensive collection of parasitic invertebrates from RefSeq which are pathogenic to humans [38]	77

**Table 9:** Statistics for generating the six Genestrip databases from Table 8 using 20 threads and a maximum allowed JVM heap size of 32 GB

DB name	Stored *k*-mers	Disk size (MB)	Wall time (min)	Max. RAM (MB)
cv_g	379,165,091	3218	47	7918
hv_g	3,865,987	32	5	1114
tb	524,530,554	3731	489	23,003
protozoa	1,332,774,884	9621	138	25,306
vineyard	688,413,684	4662	87	24,681
parasites	1,659,904,652	11,509	278	30,636

#### Read classification

To assess Genestrip’s performance regarding *k*-mer counting and read classification, we compared its analysis runs with corresponding runs under KrakenUniq, Kraken 2 und Ganon.

For the first comparison we applied the viral databases from “Classification via broad coverage databases” section to the human saliva fastq files from Table 2. Scenario (a) of Table 10 summarizes the analysis performance across all five fastq files.

In scenario (b) of Table 10 we used the bacterial databases like MicrobialDB and Standard from “Analysis databases” section vs. tb to analyze the eight tick-related fastq files from “Explorative analysis of tick-related data” section. On the one hand, comparing the tools this way may be considered “unfair” because of unequally sized databases. But for KrakenUniq, Kraken 2 and Ganon, there is no good alternative to employing broad-coverage bacterial databases for the purpose (of detecting tick-related bacterial pathogens) because, as examined in “Classification via small databases” section, a corresponding small database bares the high risk of many false positives. Since MicrobialDB is so large, we had to make use of KrakenUniq’s database processing in chunks which broke down each run into 6 rounds. This caused considerably longer run times under KrakenUniq.

In scenario (c) we had the fastq files from (b) jointly analyzed. In this case, Kraken 2 and Genestrip both benefitted considerably from loading the database just once for all eight fastq file, whereas Ganon profited only mildly and KrakenUniq not at all.

Genestrip dominates across all three scenarios with respect to classification speed.

Regarding main memory, all tools’ analysis performance is dominated by the size of the database. The required memory did not vary much across runs when the same database was used. Scenario (a) fairly compares the memory footprint of each tool since all databases where generated via the same set of genomes: As expected Kraken 2 and Ganon need less space than KrakenUniq and Genestrip because of their compressed representation of *k*-mers. Interestingly, Genestrip needs much less space than KrakenUniq still, although they both work with a similar, uncompressed representation of *k*-mers.

**Table 10:** Read analysis performance comparison between KrakenUniq, Kraken 2, Ganon and Genestrip with 10 threads for each tool

Scenario	Parameter	KrakenUniq	Kraken 2	Ganon	Genestrip
(a) Viral DBs from “Classification via broad coverage databases” section on the 5 human saliva fastq files from Table 2	Avg. reads per fastq file	824,743,085			
	Avg. BPs per read	101			
	Avg. RAM (MB)	10,617	1410	2472	4560
	Max. wall time (min)	38	40	147	37
	Avg. wall time (min)	31	34	124	30
	Avg. speed (reads/s)	442,221	402,313	110,852	458,190
(b) Broad-coverage bacterial DBs from “Analysis databases” section vs. tb on the 8 tick-related fastq files from “Explorative analysis of tick-related data” section	Min. reads per fastq file	644,767			
	Max. reads per fastq file	2,233,967			
	Avg. reads per fastq file	1,255,973			
	Avg. BPs per read	365			
	Avg. RAM (MB)	121,676	96,866	58,380	6148
	Max. wall time (min)	25.4	3.35	2.68	0.81
	Avg. wall time (min)	22.1	3.22	1.83	0.76
	Avg. speed (reads/s)	946	6508	11,523	27,303
(c) broad-coverage bacterial DBs from “Analysis databases” section vs. tb on the 8 tick-related fastq files *jointly analyzed*	RAM (MB)	121,775	97,246	58,410	6189
	Wall time (min)	180	4.8	9.61	1.5
	Speed (reads/s)	928	34,888	17,413	116,642

For MicrobialDB we set the parameter --preload-size 96G to limit KrakenUniq’s main memory according to [6]. (Otherwise, the analysis with KrakenUniq would have not been possible on the given PC)

## Conclusions

We have presented Genestrip—a bioinformatics tool for (unique) *k*-mer counting and read classification. Its major improvement over existing systems is its very low memory consumption at database generation time and at read analysis time while also maintaining very high precision and recall. To achieve this, Genestrip facilitates and suggests the generation of small databases that cover the genomes of a collection of organisms of interest. Genestrip’s update phase ensures that during database generation, the most suitable lowest common ancestor taxon is computed for each stored *k*-mer by *also considering genomes of organisms whose* *k-mers are not included in the database*. Normally, *Genestrip considers all genomes from a matching category of RefSeq for this purpose*.

The results from “Correctness” section indicate that Genestrip attains the same type of correctness as KrakenUniq in terms of database structure, *k*-mer counting and read classification given that the databases of both systems are filled via all genomes from an entire RefSeq category. But more importantly, “Classification via small databases” section highlights that Genestrip sustains high classification quality when changing from a large, comprehensive database to a small database, although just a subset of the genomes out of a RefSeq category is included in that small database. In contrast, we showed in “Small databases on real-world data” section that corresponding small databases under KrakenUniq, Kraken 2 or Ganon fail to achieve a suitable level of read classification quality for real-world data (see Table 6). This is because the latter tools do not consider genomes outside of the database to be generated like Genestrip does during its update phase at database generation time.

In “Example: Tick-borne bacterial pathogens” section has exemplified the usefulness of Genestrip’s “small databases” in the context of tick surveillance where ticks were metagenomically screened for a collection of well-known bacterial pathogens [25]. The resulting database tb has a disk size of less than 4 GB and can be used for analysis on any modern PC. In “Analysis of simulated data” section, we tested tb on simulated data that comes close to real-world data produced when screening ticks for pathogens via Nanopore technology. As opposed to typical broad coverage bacterial databases under KrakenUniq, Kraken 2 and Ganon, Genestrip combined with tb offered reasonable precision and recall in light of high error rates caused by the modeled Nanopore device (see Table 7).

The deliberate focus on a particular set of genera or species allows for more genomes to be included for the organisms of interest while the resulting Genestrip database may still remain small. In “Explorative analysis of tick-related data” section, we explored the effects of the deep database tb versus the broad coverage database MicrobialDB on the basis of real-world tick analysis data. tb for Genestrip contains almost twice as many *k*-mers from the chosen genera than MicrobialDB for KrakenUniq. Because of this, Genestrip achieved much better recall when analyzing related fastq files which is consistent with the results on simulated tick-related data from Table 7.

Regarding performance, we have examined *database generation as well as fastq file analysis*: “Database generation” section has shown that Genestrip empowers users to flexibly create new databases on regular PCs in minutes to just a few hours. As summarized in Tables 8 and 9, we created databases with different applications in mind where the generation of tb took the longest with about 8 h while main memory consumption remained below 32 GB in all of the cases.

In “Read classification” section, we examined Genestrip’s analysis performance. We compared it against KrakenUniq, Kraken 2 and Ganon by means of three scenarios where Genestrip dominated in all three cases concerning classification speed (see Table 10). In scenario (a) we used complete viral databases that were generated via the same set of genomes in all four cases and we analyzed very large short read fastq files based on human saliva. Genestrip performed best closely followed by KrakenUniq. In the scenarios (b) and (c), we applied tb from above to real-world, long read tick data and similarly, we applied broad coverage database in conjunction with the other three tools to perform the same task of detecting tick-borne pathogens. In both scenarios, Genestrip was over two times faster than any of the other analysis tools.

Regarding main memory, Genestrip’s analysis performance is dominated by the size of the database itself. E.g, memory consumption remained at about 6 GB of RAM under the database tb which has a disk size of 3.7 GB. In case of the other three analysis tools, memory consumption was also dictated by the database size. However, these tools called for broad coverage databases in scenario (b) and (c) which led to a memory consumption of 57 GB under Ganon and far more under Kraken 2 and KrakenUniq.

Unlike Kraken 2 and Ganon, Genestrip eliminates the potential for false positive *k*-mer counts because it does not compress *k*-mers under loss of information. This renders our tool suitable in fields such as medical diagnostics where precision of *k*-mer counting and read classification is paramount—which was also a motive for evolving Kraken 1 into KrakenUniq [6] after the same research group had already authored Kraken 2 [2]. However, as mentioned in “Background” section, [8] stated that generating a full bacterial database for Kraken 1 and so equivalently for KrakenUniq from RefSeq “required ca. 2.5 TB of RAM and ca. 11 days” on a suitable computer. But since then, RefSeq’s number of prokaryotic genomes alone has increased up by a factor of 5.8 to over 48 Mio. [9]. Overall, this suggests that *k*-mer databases with very broad and comprehensive coverage (such as by including *all* genomes from the RefSeq category “Bacteria”) have reached a “dead-end” when combined with lossless storing of *k*-mers. In light of this problem, different *k*-mer compressing analysis tools have been developed including Kraken 2, Ganon and others. Unfortunately, our results indicate that compression comes at the price of lowered classification quality. The drawback was evident in experiments focusing on real-world data or on simulated data with certain real-world error rates (see Tables 6 and 7 but also Table 5 of the Appendix).

Our contribution offers an alternative approach by generating small databases that focus on organisms of interest in the context of a specific application scenario. Due to this focus, *k*-mer compression becomes unnecessary. We have shown that our solution offers high classification quality and supports efficient analysis. However, as a precondition, the least common ancestor update must consider many remotely to closely related organisms whose genomes may not even be represented in the resulting database. Genestrip’s update phase from “Approach” section fulfills this demand and is incorporated in an efficient database generation procedure.

In use cases where metagenomics is meant to be performed with very few restrictions on target organisms, then Genestrip may not be the right choice. Although it is certainly possible to create large databases under Genestrip, one would end up with similar challenges as with KrakenUniq (see above). If exactness of results is required in addition to broad coverage, then a two-stage analysis procedure might prove useful: In stage one, a tool such as Kraken 2 with a compressed database for broad coverage may be applied to identify candidate organisms of interest. In stage two, Genestrip might be used to confirm and “drill down” the initial findings by potentially first generating and then using a dedicated database.

## Availability and requirements

Project name: Genestrip.

Project home page: https://github.com/pfeiferd/genestrip.

Operating system(s): Platform independent.

Programming language: Java.

Execution requirements: JRE 11 or higher.

Build requirements: JDK 11 or higher and Maven 2 or 3.

License: Apache 2.0 subject to the Commons Clause License Condition v1.0.

Any restrictions to use by non-academics: License needed for commercial use.

## Additional file

Supplementary file 1

## Data Availability

The exact code base for this publication is available under https://github.com/pfeiferd/genestrip/releases/tag/v2.5 and The code base used to generate all of the results from this publication is available under: https://github.com/pfeiferd/genestrip-db-exp/tag/v1.7 Following [40], the human saliva fastq files were acquired from the Sequence Read Archive (SRA) under the entries: ERX1462737, ERX1462740, SRX2830683, SRX2830684, and SRX2830689. Following [25], the tick-related fastq files were acquired from the Sequence Read Archive (SRA) under the entries SRX13458610, SRX13458622, SRX13458624, SRX13458626, SRX13458627, SRX13458628, SRX13458611 and SRX13458612.

## List of symbols

*t*: Placeholder for taxon
*f*: Placeholder for fastq file
*d*: Placeholder for *k*-mer database
$k_{t,f,d}$: Number of *k*-mers found for taxon *t* in fastq file *f* via database *d*
$u_{t,f,d}$: Number of unique *k*-mers found for taxon *t* in fastq file *f* via database *d*
$u_{t,d}$: Number of *k*-mers stored under taxon *t* in database *d*
cv_ku: Complete viral database for KrakenUniq
cv_g: Complete viral database for Genestrip
hv_ku: Human virus database for KrakenUniq
hv_g: Human virus database for Genestrip
FPP: False positive probability of a probabilistic filter
BPs: Base pairs
MicrobialDB: Large, comprehensive microbial database for KrakenUniq
tb: Tick-borne bacterial pathogens database for Genestrip
DB: Database
JVM: Java Virtual Machine

## Footnotes

1 By standard, RefSeq is structurally divided into 14 categories including “Bacteria”, “Fungi”, “Invertebrate”, “Protozoa”, “Viral” and others.

2 More recent probabilistic filters such as Xor filters [14] or a Ribbon filters [15] do not apply there since their construction demands all *k*-mers to be known upfront, whereas at database generation time, the *k*-mers stream in one by one from the genomic files being read.

3 Here, we chose a Blocked Bloom filter as characterized in [14] since its query performance is superior to all other filters evaluated there. The filter’s memory overhead is tolerable considering *L* and *T* together are eight times larger in bits.

4 For example, in the case of detecting infections, related samples typically contain a lot of host-DNA leading to many *k*-mers from the host in reads, whereas a related database typically would contain the pathogens *k*-mers only.

5 Considering both versions is necessary because sequenced reads may occur in either direction but also because genomes may be represented in reverse complement direction, such as in the case of circular DNA.

6 For detailed information see: https://github.com/pfeiferd/genestrip/blob/master/README.md.

7 For compatibility with KrakenUniq, we kept minKMersForClass from “Read classification” section at the default of 1.

8 See https://pirovc.github.io/ganon/default_databases/#filter-type-ibf-and-hibf.

9 The list of human viruses originates from [24]. Some human viruses known to be missing were manually added. All taxons were chosen on the species rank.

10 In this context, we do not present the results for the extracted fastq file from [21] since the file contains only 240 reads from human viruses which caused low precision outliers under KrakenUniq and Kraken 2. We therefore omit the file also below when focusing the evaluation on human viruses.

11 E.g. with a per-base error of $e_b = 0.06$, the probability for an erroneous 31-mer is $1-(1-e_b)^{31} \approx 0.85$.

12 To ensure compatibility for this experiment, we set minKMersForClass=1 under Genestrip (see “Read classification” section).

13 We ensured that as few competing processes as possible were running during the experiments and OS disk swapping was disabled. Platform details: Intel Xeon W-2255 CPU @3.70 GHz, Ubuntu 24.04.4 LTS, Java Oracle 23.0.1 64-Bit Server VM.

14 Times for downloading any of the necessary base files from the NCBI are not included. RefSeq and Genbank files were downloaded upfront via the goals refseqfna and fastasgenbankdl from Fig. 1 of the Appendix. The databases were generated via the goal db from Fig. 1 of the Appendix.
