# Supplementary material for: Genestrip: exact and efficient read classification for selected groups of organisms
Source: BMC Bioinformatics. 2026 Jun 16;27:134. doi: 10.1186/s12859-026-06512-1 (PMC13273988; doi:10.1186/s12859-026-06512-1)
Supplement: Supplementary file 1 [file 12859_2026_6512_MOESM1_ESM.pdf]

Appendix for ”Genestrip: Exact and efficient read classification for selected groups of organisms”

Daniel Pfeifer[1*], Markus Graf[1] and Clas Rurik[2]

[1*]IT Faculty, Heilbronn University, Max-Planck-Str. 39, Heilbronn, 74081, Baden-Württemberg, Germany.
[2]codeclap, Kokhaven 20, Rødekro, 6230 Denmark.

*Corresponding author(s). E-mail(s): daniel.pfeifer@hs-heilbronn.de; Contributing authors: markus.graf@hs-heilbronn.de; cru@codeclap.cc;

1 Genestrip's goal graph

Figure 1 depicts an extract of Genestrip's goal dependency graph: Several goals (prefixed with `d:`) revolve around automatically downloading all necessary files of RefSeq, Genbank and other data from the NCBI's Web site [44]. When required for database generation, the goal `refseqfna` downloads the RefSeq genomes belonging to a matching category as a collection of zip files. The collection is one of NCBI's standard offerings. By consulting the goal `checksummap`, `refseqfna` also performs an MD5 fingerprint check after each zip file download to ensure data integrity and reattempts the download in case the initial file appears to be corrupted.

Several object goals (prefixed with `o:`) load files and create data structures or compute results in main memory for further processing. Most file goals (prefixed with `f:`) write computational results to files. Boxes with edged corners depict goals that are meant to be executed directly via the command line. For example, the goal `match` is for fastq file analysis but it would trigger a respective database generation goal if the corresponding database file was missing.

Solid edges symbolize “eager” dependencies where the existence of a dependency's result is ensured prior to the execution of the dependent goal. In contrast, dotted edges symbolize “lazy” dependencies where the existence of a dependency's result is ensured when first accessed during the execution of the dependent goal.

Table 1 describes the most important goals for database generation in their typical execution order. All of these goals are multithreading-enabled for faster database generation.

Goal name	Description
`fillsize`	This goal determines the size of the database as its number of unique k-mers by reading the respective reference genome files and including the genomes of selected organisms. We employ the HyperLogLog algorithm from [46] to compute a cardinality estimate of *deduplicated*, i.e. unique, k-mers. Alternatively and for a more precise estimate, an additional temporary Bloom filter suits the same purpose.
`filldb`	This *entry phase* populates the database with all k-mers of genomes from selected organisms. The appropriate size of the database is based on `fillsize`. Duplicate k-mer entries are avoided via a Bloom filter that tracks the already stored k-mers. Finally, the stored k-mers are sorted in order to enable binary search at analysis time but also for the goal `updatedb` from below.
`updatedb`	This *update phase* updates the populated database to account for common k-mers between organisms. *All* genomes from RefSeq belonging to a set of pre-configurable taxonomic categories are considered during this phase. This ensures that a k-mer from a selected organism obtains the correct taxon as the k-mer may also occur in the genome of another organism (which might be represented in the database or not). In the finished database, the respective k-mer is associated with the lowest common ancestor taxon as computed via all considered genomes that contain the k-mer.

Table 1 Genestrip's most relevant goals for database generation

Besides k-mer matching and read classification, Genestrip supports filtering of fastq files via a probabilistic filter in similarity to [45]. When generated via the goal `index`, the filter gets stored in a separate file. The goal `filter` loads the filter and processes fastq files accordingly. The standard configuration of the filtering process rewrites a read to the output if the read contains at least one k-mer that is recognized by the filter as potentially belonging to a selected organism of interest.

2 The dust function d

We designed a simple dust function $d(s)$ that allows for recognizing and potentially excluding highly repetitive k-mers s based on the following rationale:

1. Simple repeats like `TTTTTT`, repeating pairs like `ATATAT` and repeating triplets like `ATCATC` should be recognized. (Triplets also form the basis of the approach in [18].)
2. The function should be based on the number of times a single base, a pair or a triplet is repeated. E.g. `AT` is repeated twice in `ATATAT` and `ATC` is repeated once in `ATCATC`.
3. A repeat's contribution to d should grow more than linearly with the repeat's length. E.g., `AAAACA` should obtain at least the d-value of `AACAAA` (or more).
4. d should be symmetrical. This is because a k-mer and its reverse complement should obtain the same dust value [18].
5. $d(s)$ should be computable in $O(|s|)$ and should lend itself for streaming computation. I.e., after $d(s) = d(s_1 \dots s_k)$ has been computed, $d(s') = d(s_2 \dots s_k s'_{k+1})$ should be computable in constant time.

To specify the dust function d, we first define the *skip-c-repeat length* at position i in a string s where $1 \leq i \leq |s|$, $c \geq 0$:

$$srl_c(s,i) = \max\{r \mid i+r-1 \leq |s|-c-1 \wedge \bigwedge_{j=i}^{i+r-1} s_j = s_{j+c+1}\}$$

E.g., two skip-1-repeat lengths for $s = \texttt{ACTCT}$ are $srl_1(s,1) = 0$ and $srl_1(s,2) = 2$. Note that r's range is confined to $0 \leq r \leq |s| - c - i$.

We then determine the *next position* $np_c(s,l)$ of the l-th skip-c-repeat length in s with $|s| \geq 1$ as follows:

$$np_c(s,l) = \begin{cases} 1 & \text{for } l = 1 \\ np_c(s,l-1) + srl_c(s, np_c(s,l-1)) + 1 & \text{for } l > 1, np_c(s,l-1) < |s| \end{cases}$$

E.g. for $s = \texttt{ACACTC}$ we get $np_1(s,1) = 1$ and $np_1(s,2) = 4$. For clearer discussion, we declare a *skip-c-repeat* to be a substring of s of length $srl_c(s,i)$ at a position i *as provided by* np_c (but not at any other positions).

The dust function $d(s)$ sums over the lengths of all skip-c-repeats in s with $c = 0, \ldots, c_{\max}$ transformed via a variant of the Fibonacci function [48] where $fib(0) = 0, fib(1) = 1, fib(2) = 2$ and $fib(n) = fib(n-1) + fib(n-2)$ for $n > 2$:

$$d(s) = \sum_{c=0}^{c_{\max}} \sum_{l=1}^{np_c(s,l) \leq |s|} fib(srl_c(s, np_c(s,l)))$$

The use of the Fibonacci function in d accounts for point 3 from the above rationale.

$d(s)$ is symmetrical: Let $s' = s_k \ldots s_1$ be the reverse of $s = s_1 \ldots s_k$; then it follows from the definition of srl_c that for a skip-c-repeat at position i, there is $srl_c(s,i) = srl_c(s', |s| - i - srl_c(s,i) + 1)$. Since $np_c(s,l)$ determines the positions of all skip-c-repeats, doing so in reverse order leads to the same skip-c-repeat lengths and so, $d(s) = d(s')$.

$d(s)$ does not compensate for double counts, e.g. $srl_0(\texttt{AAAA}, 1) = 3$ but also $srl_1(\texttt{AAAA}, 1) = 1$. We consider this as legitimate since AAAA is indeed both a skip-0-repeat of A and a skip-1-repeat of AA. In the general case, given some skip-c-repeat h and a multiple $f+1$ of $c+1$ with $f \leq c_{\max}$: If $|h| > f$, then h is also a skip-f-repeat. Therefore, h gets "boosted" in d via any corresponding multiple $f+1$.

For an efficient implementation that considers no more but triplets, we set $c_{\max} = 2$. Although the functional specification of $d(s)$ from above may seem intricate, its computation is simple and iterates just once over s. Listing 1 presents a basic Java implementation that computes $d(s)$ for $c_{\max} = 2$.[1] It confirms the desired complexity of $O(|s|)$ given that $c_{\max}$ is constant. With regard to Genestrip, we implemented a streaming variant as well with the qualities as described in the rationale from above (not shown).

[1]Although the presented algorithm works just for $c_{\max} = 2$, it can obviously be generalized for a larger $c_{\max}$ by introducing appropriate loops over $c = 0, \ldots, c_{\max}$.

Listing 1 Computation of $d(s)$ for $c_{\max} = 2$ in Java with a simplistic representation of a string s.

```
// int fib[] = ... is assumed to be defined beforehand.
public int d(byte[] s) {
    int d = 0, srl0 = 0, srl1 = 0, srl2 = 0; // skip-c-repeat lengths
    int l1 = -1, l2 = -1, l3 = -1;           // previous elements in s
    for (int i = 0; i < s.length; i++) {
        if (s[i] == l1) srl0++;
        else {
            d += fib[srl0];
            srl0 = 0; }
        if (s[i] == l2) srl1++;
        else {
            d += fib[srl1];
            srl1 = 0; }
        if (s[i] == l3) srl2++;
        else {
            d += fib[srl2];
            srl2 = 0; }
        l3 = l2; l2 = l1; l1 = s[i];
    }
    d += fib[srl0] + fib[srl1] + fib[srl2];
    return d;
}
```

Table 2 shows examples of 31-mers for $c_{\max} = 2$ at different thresholds t with $d(s) \geq t$. Figure 2 shows the ratio of 31-mers selected via $d(s) \geq t$ for $c_{\max} = 2$ when increasing the threshold t.

t	s	$d(s)$
1	GCCTGACTGACTGACTGACAGCTTGACTGAC	4
5	GATCGAATCGCATGCGTACGTATGCCTAGCT	5
10	GACTCGAATTCATTCGAAGCATCGATTGACT	10
50	CCACTAAAAAAATGTTTTTCGCAGTGATGAT	50
100	TACAAGGCTTGATTCTGATATATATATATGA	100
500	TTTTTTTTTTTTTCGGTGTTTTTTTGTTACT	502
1000	TGCGAAAGCGCGCGCGCGCGCGCGAGGGCGG	1000
5000	CACTAGCCCCCCCCCCCCCCCCCCGCCTGTC	5175

Table 2 Example 31-mers s for $c_{\max} = 2$ at different thresholds t with $d(s) \geq t$. The 31-mers were randomly drawn and then selected to be close to the corresponding threshold t.

3 The *k*-mer consistency ratio

Genestrip introduces a novel, derived statistical measure, the *k-mer consistency ratio*, as explained in the following: Given $k_{t,f,d}$, the apriori probability $p(t \mid k_{t,f,d})$ of a

unique k-mer from d belonging to t to occur *at least once* in f is approximately:[2]

$$p(t \mid k_{t,f,d}) = 1 - (1 - 1/u_{t,d})^{k_{t,f,d}} \tag{1}$$

The corresponding *expected number of unique k-mers* is as follows:

$$E(k_{t,f,d}, u_{t,d}) = u_{t,d} \cdot p(t \mid k_{t,f,d}) \tag{2}$$

It is useful to compare $E(k_{t,f,d}, u_{t,d})$ with the actual count $u_{t,f,d}$: If these quantities divert too much from each other, then the consistency of the k-mer counting results for t in f should be questioned. If on the other hand, these quantities are close, then this supports the consistency of the counting results. This leads to the *k-mer consistency ratio*, which ideally is close to 1:[3]

$$r_{t,f,d} := u_{t,f,d}/E(k_{t,f,d}, u_{t,d}) \tag{3}$$

Table 3 gives a brief, real-world example where $r_{t,f,d}$ proves useful.

t	$u_{t,d}$	$k_{t,f,d}$	$u_{t,f,d}$	$E(k_{t,f,d}, u_{t,d})$	$r(k_{t,f,d}, u_{t,f,d})$	Coverage $u_{t,f,d}/u_{t,d}$
10376	139502	4151610	120419	139514	0.8631	0.8632

Table 3 Real extract of Genestrip analysis results for Human Gammaherpesvirus 4 from sequencing a human saliva sample: The k-mer count is over 34 times higher than the unique k-mer count but due to the short viral genome this is still close to the expectation as indicated by the high k-mer consistency score.

$E(k_{t,f,d}, u_{t,d})$ is included in Genestrip's analysis reports as a column named `exp. unique kmers`. The k-mer consistency ratio $r_{t,f,d}$ is represented via the column `unique kmers / exp.`.

4 Details regarding correctness

The scatter plot in Figure 3 visualizes the identical structure of the two complete viral databases created via KrakenUniq and Genestrip with 379,181,604 k-mers in total.

[2]The approximation is based on the assumption that all k-mers in d belonging to t are equally likely to be found in f (such as reported in [25, Fig. 3]). However, this assumption does not hold for k-mers with high duplicity in genomes (such as caused by genomic dust) or when special DNA library preparation protocols (such as 16S or 18S amplification) are used [41, 42]. In these cases, the k-mer consistency ratio should therefore be disregarded.

[3]A mathematically sounder approach is to consider the probability distribution $q(u_{t,f,d} \mid k_{t,f,d})$, i.e., the probability of obtaining exactly $u_{t,f,d}$ unique k-mers for a given (non-unique) k-mer count of $k_{t,f,d}$ by sampling with replacement as the underlying random process. Interestingly, this distribution is non-trivial and has been investigated in [43]. The authors have shown that the distribution can be approximated by a normal distribution $\mathcal{N}(E(k_{t,f,d}, u_{t,d}), \sigma_q^2)$ under most practically relevant conditions. Moreover, they confirm the expectation $E(k_{t,f,d}, u_{t,d})$ from above and give a formula for the variance σ_q^2 which in our case, depends on $k_{t,f,d}$ and $u_{t,d}$. Although mathematically sound, the approach did not prove useful with respect to k-mer counts because $\mathcal{N}$ tends to be extremely peaked and so, even small deviations from $E(k_{t,f,d}, u_{t,d})$ result in a $q(u_{t,f,d} \mid k_{t,f,d}) \approx 0$.

As real-world examples, we applied the two viral databases to short-read fastq files from DNA-sequencing human saliva of five different donors according to Table 2 of the main document. To visualize the identity of the results, Figure 4 shows scatter plots of respective k-mer counts and read counts from KrakenUniq and Genestrip for one of the fast files.

5 Additional results regarding small databases

The additional results include Table 4 and Table 5.

System	Data	Species			Genus		
		**Prec.**	**Recall**	**F_1**	**Prec.**	**Recall**	**F_1**
KrakenUniq	MiSeq	0.96	0.96	0.96	0.96	0.96	0.96
Kraken 2 (S)	MiSeq	0.96	0.96	0.96	0.96	0.96	0.96
Kraken 2 (C)	MiSeq	0.92	0.90	0.91	0.95	0.93	0.94
Ganon (S)	MiSeq	0.96	0.96	0.96	0.96	0.96	0.96
Ganon (C)	MiSeq	0.96	0.80	0.87	0.96	0.80	0.87
Genestrip (S)	MiSeq	0.96	0.96	0.96	0.96	0.96	0.96
Genestrip (C)	MiSeq	0.96	0.96	0.96	0.96	0.96	0.96
KrakenUniq	HiSeq	0.97	0.97	0.97	0.98	0.98	0.98
Kraken 2 (S)	HiSeq	0.97	0.97	0.97	0.98	0.98	0.98
Kraken 2 (C)	HiSeq	0.95	0.94	0.95	0.98	0.96	0.97
Ganon (S)	HiSeq	0.97	0.97	0.97	0.98	0.98	0.98
Ganon (C)	HiSeq	0.97	0.92	0.95	0.98	0.93	0.95
Genestrip (S)	HiSeq	0.97	0.97	0.97	0.98	0.98	0.98
Genestrip (C)	HiSeq	0.97	0.97	0.97	0.98	0.98	0.98

Table 4 Read classification quality for simulated fastq files from "Correctness" section of the main document analyzed with KrakenUniq, Kraken 2, Ganon and Genestrip using the complete viral databases from "Classification via broad coverage databases" section of the main document but with an evaluation focus on human viruses only. The systems' adaptions for "(S)" and "(C)" are based on Table 3 of the main document.

6 Additional results regarding tick-related analysis

The additional results can be found in Table 6.

System	Base file name	Species			Genus		
		Prec.	Recall	F_1	Prec.	Recall	F_1
KrakenUniq	SRR5571985	0.04	0.92	0.07	0.04	0.92	0.07
Kraken 2 (S)	SRR5571985	0.03	0.65	0.06	0.03	0.65	0.07
Kraken 2 (C)	SRR5571985	0.10	1.00	0.19	0.10	1.00	0.19
Ganon (S)	SRR5571985	0.02	0.66	0.03	0.02	0.66	0.03
Ganon (C)	SRR5571985	0.10	1.00	0.18	0.10	1.00	0.18
Genestrip (S)	SRR5571985	0.50	1.00	0.67	0.50	1.00	0.67
Genestrip (C)	SRR5571985	0.91	1.00	0.95	0.91	1.00	0.95
KrakenUniq	ERR1395613	0.04	0.92	0.07	0.04	0.92	0.07
Kraken 2 (S)	ERR1395613	0.03	0.66	0.06	0.03	0.66	0.06
Kraken 2 (C)	ERR1395613	0.31	1.00	0.47	0.31	1.00	0.47
Ganon (S)	ERR1395613	0.02	0.69	0.04	0.02	0.70	0.04
Ganon (C)	ERR1395613	0.28	1.00	0.44	0.28	1.00	0.44
Genestrip (S)	ERR1395613	0.50	1.00	0.67	0.50	1.00	0.67
Genestrip (C)	ERR1395613	0.90	1.00	0.95	0.90	1.00	0.95
KrakenUniq	SRR5571991	0.04	0.91	0.07	0.04	0.91	0.07
Kraken 2 (S)	SRR5571991	0.03	0.65	0.06	0.03	0.65	0.06
Kraken 2 (C)	SRR5571991	0.40	1.00	0.57	0.40	1.00	0.57
Ganon (S)	SRR5571991	0.02	0.69	0.04	0.02	0.70	0.04
Ganon (C)	SRR5571991	0.25	1.00	0.40	0.25	1.00	0.40
Genestrip (S)	SRR5571991	0.50	1.00	0.67	0.50	1.00	0.67
Genestrip (C)	SRR5571991	0.93	1.00	0.96	0.93	1.00	0.96
KrakenUniq	ERR1395610	0.04	0.93	0.08	0.04	0.93	0.08
Kraken 2 (S)	ERR1395610	0.04	0.72	0.08	0.04	0.72	0.08
Kraken 2 (C)	ERR1395610	0.60	1.00	0.75	0.60	1.00	0.75
Ganon (S)	ERR1395610	0.02	0.72	0.05	0.02	0.72	0.05
Ganon (C)	ERR1395610	0.57	1.00	0.73	0.57	1.00	0.73
Genestrip (S)	ERR1395610	0.55	1.00	0.71	0.55	1.00	0.71
Genestrip (C)	ERR1395610	0.95	1.00	0.97	0.95	1.00	0.97
KrakenUniq	SRR5571990	0.04	0.91	0.07	0.04	0.91	0.07
Kraken 2 (S)	SRR5571990	0.03	0.64	0.06	0.03	0.64	0.06
Kraken 2 (C)	SRR5571990	0.01	1.00	0.02	0.01	1.00	0.02
Ganon (S)	SRR5571990	0.02	0.67	0.04	0.02	0.67	0.04
Ganon (C)	SRR5571990	0.15	1.00	0.27	0.15	1.00	0.27
Genestrip (S)	SRR5571990	0.50	1.00	0.67	0.50	1.00	0.67
Genestrip (C)	SRR5571990	0.92	1.00	0.96	0.92	1.00	0.96

Table 5 Read classification quality for the the real-world fastq files from Table 2 of the main document analyzed with KrakenUniq, Kraken 2, Ganon and Genestrip with an evaluation focus on human viruses only. The evaluation compares the classifications when using a "human virus only" database against each system's own classifications obtained when using the corresponding complete viral database from "Classification via broad coverage databases" section of the main document. The systems' adaptions for "(S)" and "(C)" are based on Table 3 of the main document.

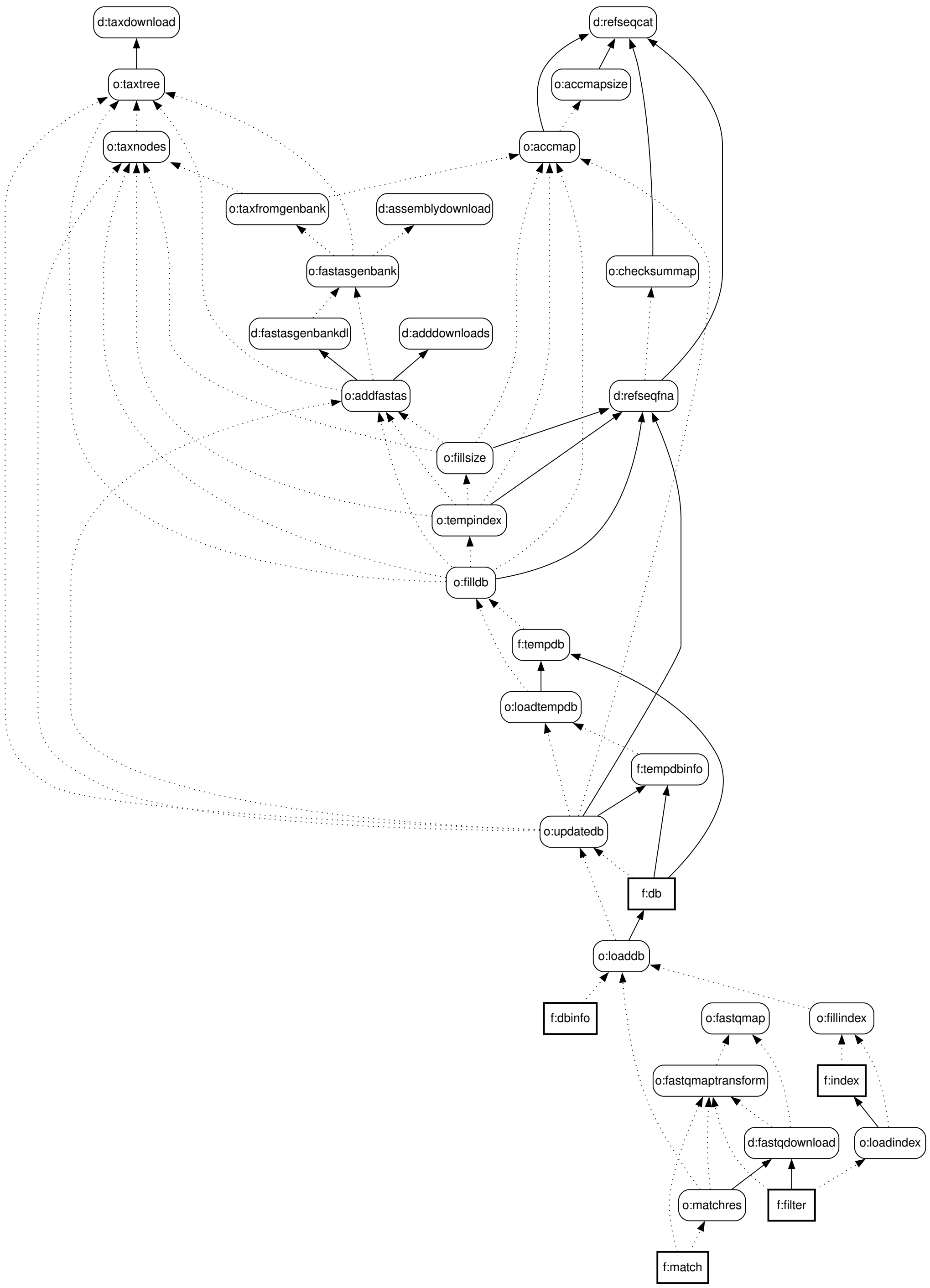

Fig. 1 Extract of Genestrip's goal dependency graph

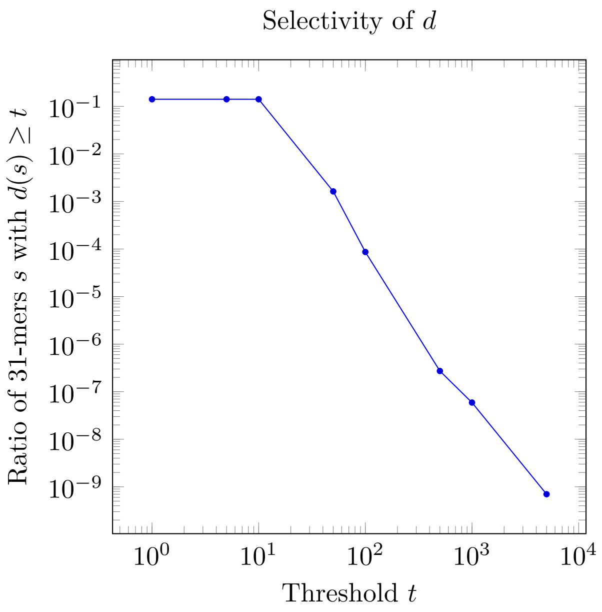

Fig. 2 The ratio of 31-mers s selected via $d(s) \geq t$ for $c_{\max} = 2$ when increasing the threshold t (based on a logarithmic scale for both axes). The data points are based on the t-values from Table 2 and were estimated via 10.000.000.000 uniform draws over all 31-mers.

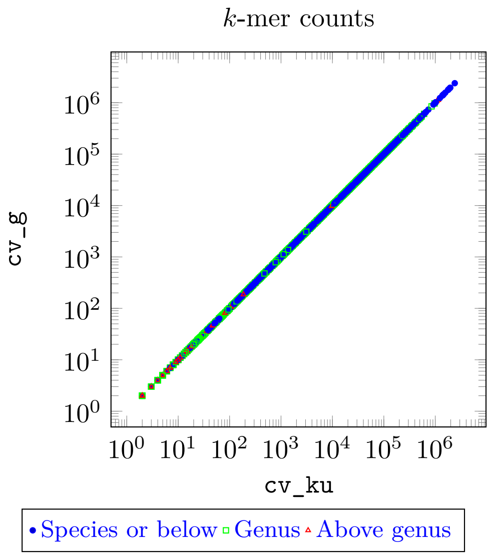

Fig. 3 Scatter plot of k-mer counts for the RefSeq databases `cv_ku` from KrakenUniq and `cv_g` from Genestrip covering the entire category "Viral" (16,513 data points, based on a logarithmic scale with 1 added to each value in order to enable the representation of zero-counts).

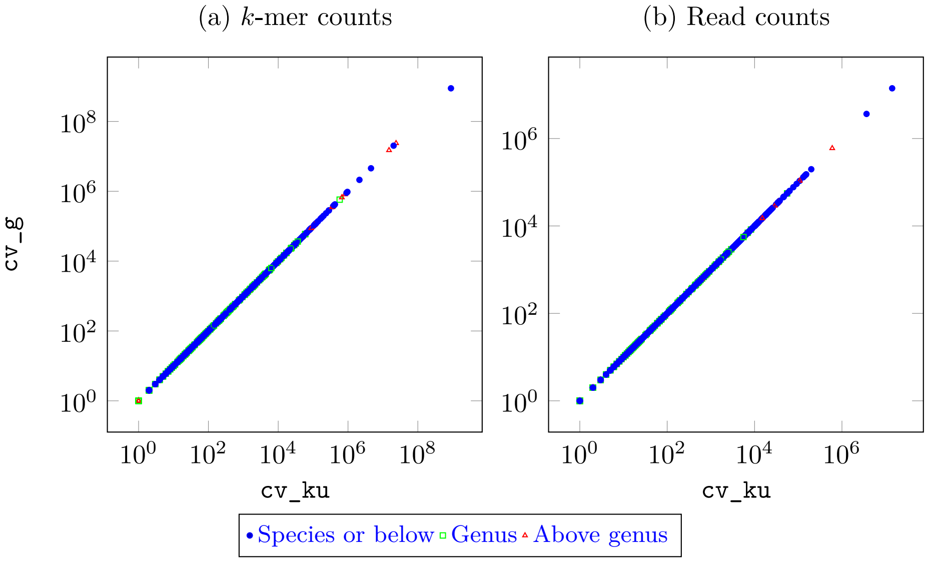

Fig. 4 Scatter plots of k-mer counts (a) and read counts (b) for fastq file #3 from Table 2 of the main document using the complete viral databases from Figure 3 (1,851 data points, based on a logarithmic scale with 1 added to each value to enable the representation of zero-counts).

System	Data	Species Prec.	Species Recall	Species F_1	Genus Prec.	Genus Recall	Genus F_1
KrakenUniq	Tick 1	0.94	0.65	0.77	1.00	0.69	0.81
Kraken 2 (S)	Tick 1	0.94	0.61	0.74	1.00	0.65	0.78
Kraken 2 (C)	Tick 1	0.77	0.00	0.00	1.00	0.00	0.00
Ganon (S)	Tick 1	0.84	0.61	0.71	0.90	0.65	0.75
Ganon (A)	Tick 1	0.63	0.63	0.63	0.67	0.67	0.67
Genestrip (S)	Tick 1	0.89	0.67	0.76	0.97	0.73	0.83
Genestrip (C)	Tick 1	0.88	0.63	0.73	0.96	0.69	0.80
KrakenUniq	Tick 2	0.17	0.03	0.05	1.00	0.19	0.32
Kraken 2 (S)	Tick 2	0.30	0.09	0.14	1.00	0.32	0.48
Kraken 2 (C)	Tick 2	0.16	0.00	0.00	1.00	0.00	0.00
Ganon (S)	Tick 2	0.23	0.09	0.13	0.64	0.25	0.36
Ganon (A)	Tick 2	0.09	0.09	0.09	0.25	0.25	0.25
Genestrip (S)	Tick 2	0.42	0.30	0.35	0.74	0.54	0.62
Genestrip (C)	Tick 2	0.40	0.26	0.32	0.72	0.47	0.57
KrakenUniq	Tick 3	0.65	0.18	0.28	1.00	0.27	0.43
Kraken 2 (S)	Tick 3	0.64	0.22	0.33	1.00	0.35	0.52
Kraken 2 (C)	Tick 3	0.24	0.00	0.00	1.00	0.00	0.00
Ganon (S)	Tick 3	0.50	0.22	0.30	0.69	0.31	0.43
Ganon (A)	Tick 3	0.22	0.22	0.22	0.31	0.31	0.31
Genestrip (S)	Tick 3	0.58	0.44	0.50	0.75	0.57	0.65
Genestrip (C)	Tick 3	0.56	0.40	0.47	0.74	0.52	0.61
KrakenUniq	Tick 4	0.29	0.04	0.07	1.00	0.13	0.23
Kraken 2 (S)	Tick 4	0.46	0.10	0.17	1.00	0.23	0.37
Kraken 2 (C)	Tick 4	0.28	0.00	0.00	1.00	0.00	0.00
Ganon (S)	Tick 4	0.24	0.08	0.12	0.53	0.17	0.26
Ganon (A)	Tick 4	0.08	0.08	0.08	0.17	0.17	0.17
Genestrip (S)	Tick 4	0.48	0.32	0.38	0.67	0.45	0.54
Genestrip (C)	Tick 4	0.45	0.28	0.34	0.64	0.39	0.49
KrakenUniq	Tick 5	0.27	0.03	0.06	1.00	0.12	0.21
Kraken 2 (S)	Tick 5	0.48	0.10	0.17	1.00	0.21	0.34
Kraken 2 (C)	Tick 5	0.37	0.01	0.01	1.00	0.02	0.04
Ganon (S)	Tick 5	0.20	0.06	0.10	0.46	0.14	0.22
Ganon (A)	Tick 5	0.06	0.06	0.06	0.14	0.14	0.14
Genestrip (S)	Tick 5	0.60	0.43	0.50	0.75	0.54	0.63
Genestrip (C)	Tick 5	0.58	0.39	0.46	0.73	0.49	0.59

Table 6 Read classification quality for simulated data derived from an analysis of bacterial pathogens in ticks [25]. The results are based on five simulated fastq files generated using NanoSim [35, 36] on the basis of five corresponding real-world, tick-related fastq files. "Analysis databases" section of the main document describes the databases used and "Analysis of simulated data" section of the main document discusses the analyzed data. The systems' adaptions for "(S)" and "(C)" are based on Table 3 (main document); "Ganon (A)" is described in "Analysis of simulated data" section (main document).
